# Supplementary material for: Heat exchange characteristics of underground and pavement buried pipes for bridge deck heating conditions
Source: PLoS One. 2024 May 14;19(5):e0298077. doi: 10.1371/journal.pone.0298077 (PMC11093364; doi:10.1371/journal.pone.0298077)
Supplement: S1 File — (DOCX) [file pone.0298077.s001.docx]

The determination of heating requirement for snow/ice removal, which is normally described by surface heat flux, provides important design capacity of the whole system. The required heat flux for snow melting on the basis of steady-state energy balance is given:

$q_{0}=q_{1}+q_{2}+q_{3}+q_{4}-q_{5}$ (A1)

where, *q*_0_ denotes to heat flux required at snow melting surface, W/m^2^; *q*_1_ is the heat flux required to raise the temperature of snow/ice, W/m^2^:

$q_{1}=\rho_{w}S\left[C_{w}(t_{0}-0)+C_{snow}\left(0-t_{a}\right)\right]$ (A2)

*q*_2_ is the latent heat of snowmelt W/m^2^:

$q_{2}=\rho_{w}\times S\times H_{if}$ (A3)

*q*_3_ is the long-wave radiant heat, W/m^2^:

$q_{3}=h_{c}(t_{0}-t_{a})+\varepsilon C_{b}\left[\left(\frac{t_{\text{a}}+273}{100}\right)^{4}-\left(\frac{t_{a}+273}{100}\right)^{4}\right]$ (A4)

*q*_4_ is the heat flux required for convection heat transfer, W/m^2^:

 (A5)

*q*_5_ is the solar radiant heat, W/m^2^:

$q_{5}=\alpha I$ (A6)

The concepts and the values of the above symbols are listed in Table 1.

Table 1 Concepts and the values of the snow melt calculation parameters for Jinan City

| Symbols | Concepts | Values | Unit |
| --- | --- | --- | --- |
| *ρ*_w_ | Water density | 1000 | kg/m^3^ |
| *S* | Snowfall speed | 2.5 | mm/h |
| *C*_w_ | Specific heat of water | 4.2 | KJ/(kg·℃) |
| *C*_snow_ | Specific heat of snow | 2.1 | KJ/(kg·℃) |
| *t*_0_ | The temperature of the snow water | 0 | ℃ |
| *t*_a_ | Air temperature | -3.2 | ℃ |
| *H*_if_ | Latent heat of snowmelt | 334 | KJ/kg |
| *h*_c_ | Convective heat transfer coefficient | 17.79 | W/(m^2^⋅K) |
| *C*_b_ | Stefan-Boltzmann | 5.67 | W/(m^2^⋅K^4^) |
| *ε* | Pavement emissivity for asphalt pavement | 0.96 | - |
| *H*_f_ | Latent heat of vaporization of water | 2502c | KJ/kg |
| *ϕ* | Relative humidity of the air | 45 | % |
| *ρ*_wa_ | Saturated water vapor density at 0 ℃ | 0.00485 | kg/m^3^ |
| *h*_m_ | The rate of mass transfer when water evaporates | 0.68 | m/s |
| *α* | Surface solar radiation absorption | 0.6 | - |
| *I* | The amount of solar radiation on the Earth's surface | 182.52 | W/m^2^ |
