# Supplementary material for: Heat exchange characteristics of underground and pavement buried pipes for bridge deck heating conditions
Source: PLoS One. 2024 May 14;19(5):e0298077. doi: 10.1371/journal.pone.0298077 (PMC11093364; doi:10.1371/journal.pone.0298077)
Supplement: Attachment — Submitted filename: PONE.docx [file pone.0298077.s002.docx]

**Comments to the author**

The paper provides a clear overview on the heat exchange characteristics of underground and pavement buried pipes for bridge deck heating conditions. However, the paper sets several drawbacks which should be incorporated before final publication

Abstract: Revise the abstract carefully. Which finite element software has been employed in the present study? Mention the findings of the study.

The abstract mentions “The influences of the inlet temperature, backfill material, drilling depth, and hole spacing on the heat transfer efficiency of the ground heat exchanger were studied under the alternative engineering conditions”. What are the alternative engineering conditions mentioned here? Explain them.

Keywords: Arrange the keywords in alphabetical order.

Introduction: Elaborate the introductory part.

It is written that “Many factors affect the thermal performance of ground heat exchanger (GHE), so several studies have been conducted from different perspectives”. What are those factors? Explain them.

Mention objectives and research significance of the study.

What is novelty in the present study?

Figure 2 shows Three-dimensional geometric model and the meshing of buried U-type pipe. How the meshing was achieved?

For explaining discretization and meshing of pipeline, it’s good to cite these very recently published papers in this study.

[Blast performance enhancement prediction of circular column with helical mesh reinforcement and strengthened with UHPFRC plaster, and CFRP wrapping under close-in blast](https://scholar.google.com/citations?view_op=view_citation&hl=en&user=kp6wWdEAAAAJ&citation_for_view=kp6wWdEAAAAJ:UeHWp8X0CEIC); R Tahzeeb, M Alam, SM Muddassir; International journal of structural engineering, 1-38.

[Dynamic response of CFST column with in-plane cross reinforcement and partial CFRP wrapping upon contact blast](https://scholar.google.com/citations?view_op=view_citation&hl=en&user=kp6wWdEAAAAJ&citation_for_view=kp6wWdEAAAAJ:YsMSGLbcyi4C). R Tahzeeb, M Alam, SM Anas, SM Muddassir; Innovative Infrastructure Solutions 8 (9), 241

In section 2.2, it is written that “a controlled variable research method was employed,”. How this controlled variable methodology was achieved?

Check the citations of the Figures and Tables carefully.

Elaborate the Field test and result.

The result and conclusion parts are very brief. There are only 3 findings and only 1^st^ finding is relevant. 2^nd^ and 3^rd^ findings are part of result, they don’t state any relevant information. The conclusion must include at least 3 findings.
