# Supplementary material for: Heat exchange characteristics of underground and pavement buried pipes for bridge deck heating conditions
Source: PLoS One. 2024 May 14;19(5):e0298077. doi: 10.1371/journal.pone.0298077 (PMC11093364; doi:10.1371/journal.pone.0298077)
Supplement: Attachment — Submitted filename: Response to Reviewers.docx [file pone.0298077.s003.docx]

Reply to reviewers’ comments

The authors gratefully acknowledge the constructive comments from the Reviewers. The revised manuscript was prepared in order to address all the comments, as described in details below. Major modifications are highlighted in the revised manuscript using yellow color for easy reference.

Reviewers #1

**Comment 1.** *1. Abstract: After reading the abstract of the article, I find that there is no clear understanding of why you conducted this research. A good abstract should include the scientific problem/challenge, which I don't see in the first 2-3 sentences of your abstract. Please, explore the abstracts of several good articles - they usually consist of approximately 30% of Background, 30% of Methodology, and 40% of Key Findings. I want to emphasize that by "key findings," I mean the key results of your study that provide valuable knowledge to the scientific community, along with the main reasons why GSHPS yielded these particular results. As it stands, your abstract has not captured the reader's interest.*

*So far, in your Abstract I see only a methodology that does not reflect the specifics..*

**Reply**:

Thank you very much for your constructive comments, we improved our writing and revised the abstract accordingly as shown below:

“**Abstract:** Geothermal energy is increasingly employed across diverse applications, with bridge deck snow melting emerging as a notable utilization scenario. In Jinan city, China, a project is underway to utilize ground source heat pumps (GSHPS) for heating bridges. However, essential operational parameters, including fluid medium, temperature, and heat exchange details, are currently lacking. This study addresses the thermal design challenges associated with ground heat exchangers (GHE) for bridge heating through a combination of numerical modeling and field experiments. ~~Unlike traditional utilization of ground source heat pump system (GSHPS) on buildings, bridge heating using geothermal energy in winter have special requirements for fluid media, temperature, and heat extraction.~~ ~~Firstly, based on~~ ~~an engineering case of snow melting bridge deck with GSHPS constructed in Jinan China, A three-dimensional heat transfer model using computational fluid dynamics was built and verified.~~ ~~Then, the controlled variable research method was employed to learn the thermal performance.~~ ~~The influences of the inlet temperature, backfill material, drilling depth, and hole spacing on the heat transfer efficiency of the ground heat exchanger were studied under the alternative various engineering conditions~~. ~~Based on on-site construction testing, the influence of pipeline spacing on bridge deck temperature rise and the temperature variation characteristics of backfill materials were obtained studied~~. Utilizing software Fluent, a refined three-dimensional multi-condition heat transfer numerical analysis was carried out. Field tests based on actual operating conditions were also conducted and the design parameters were verified. The results indicate that an inlet temperature of 5°C and an aqueous solution of ethylene glycol with a mass concentration of 35% as the heat exchange medium are suitable for the GSHPS in Jinan; Moreover, the influence of backfill material and operation time on the heat transfer efficiency was revealed and the suitable material with 10% bentonite and 90% SiO2 was suggested; Finally, based on the influence of the pipe spacing on the heating characteristics of bridge deck, the transition spacing of 0.2m is given for the temperature response of the bridge deck. This comprehensive study contributes valuable insights through simulation and experimental analysis of the thermal environment variation, aiming to advance the development of GSHPS for bridge deck heating in Jinan, China.

”

**Comment 2.** *Introduction: In the Introduction section, the impression is given that the GSHPS field has already been thoroughly studied and holds little scientific interest. I strongly recommend that the authors clarify the limitations of previous research in this area to highlight the significance of their own work.*

*The authors rely on literature from 2007-2012 to describe the 'latest scientific advancements,' but it is my understanding that many developments have occurred in the field in recent years. I strongly suggest that the authors consult more recent literature to provide a more current context for their work. For example, what about phase change materials that are used in soil for thermal stabilization? Or thermosyphons? A comprehensive literature review is advised.*

**Reply**:

Thanks to your comments, we have revised the introduction to improve its taste of science and reviewed more current context as suggested. Some revisions are as

“Due to the unfriendly carbon emissions of fossil fuels, the demand for sustainable new energy is gradually increasing. As an effective and green energy source, geothermal energy is gradually used in a variety of building facilities of the world [1-3]. The thermal energy from the ground can be utilized as a heat source in heat pumps (HPs) to reduce the rapid consumption of fossil fuels. the systems of ground source heat pumps GSHPS, utilizing the geothermal energy are known as geothermal heating pumps that absorb/transfer thermal energy to/from the earth using a series of buried pipes and which can be considered one of the most efficient technologies in the heating/cooling industry. Over the last few decades, GSHPS, because of the high-performance factor and more economical operating costs, have acquired significant attention as sustainable alternative energy resources for building cooling/heating applications [4]. ~~To further improve the geothermal utilization in heating system for bridge decks, it is crucial to study the heat transfer capacity of the heat exchangers and obtain the optimal parameters governing its performance.~~

The first interest in the technology of GSHP was known after the Second World War until the early 1950s in North America and Europe. Another significant surge occurred in the 1970s following the global oil crisis, marking a period of increased GSHP activity worldwide.

……

Bridge deicing is critical to ensure roadway safety, mobility, and productivity. Adding salts to melt ice/snow are the most common deicing method but are corrosive to the bridge surfaces and polluting to environment.

……

Previous studies concluded the inlet temperature and flow rate of medium, the thermal conductivity of backfill material, the borehole depth and spacing having different effects on the engineering performance of GSHPS. Although some experimental and simulation analysis on GSHPS had been conducted, ~~due to the different types of pipe, backfill materials and stratum properties,~~ theoretical guidance on the design of GHE with the bridge deck heating conditions has not been unified. To further improve the geothermal utilization in heating system for bridge decks under the specific geological and atmospheric conditions for Jinan of China, it is crucial to study the heat transfer capacity of the heat exchangers and obtain the optimal parameters governing its performance.

”

Some current works:

[1] Hussein M. Maghrabie, Mahrousa M. Abdeltwab, Mohamed Hamam M. Tawfik. Ground-source heat pumps (GSHPs): Materials, models, applications, and sustainability, *Energ. Buildings*, 2023, 299: 113560,
[2] Chen, X.; Kong, G.; Liu, H.; Yang, T.; Zhu, X. Experimental on thermal performance of bridge deck with hydronic heating system. *Cold Reg Sci Technol*, 2020, 178(3): 103130.
[3] Yu, X.; Hurley, M. T.; Li, T.; Lei, G.; Pedarla, A. Puppala, A.J. Experimental feasibility study of a new attached hydronic loop design for geothermal heating of bridge decks. *Appl Therm Eng*, 2020(164):114507
[4] Liu, X.B.; Rees, S.J.; Spitler. J.D. Modeling snow melting on heated pavement surfaces. Part I: Model development. Appl. Therm. Eng., 2007, 27 (5–6): 1115-1124,
[5] Ho, I. H.; Li, S.; Abudureyimu, S. Alternative hydronic pavement heating system using deep direct use of geothermal hot water. *Cold Reg Sci Technol,* 2019. 160(APR.): 194-208.
[6] Lei, G. , Yu, X. , Li, T. , Habibzadeh-Bigdarvish, O. , & Hurley, M. T. . (2022). Insulated pex-pipe loops for deicing on existing bridge deck using geothermal energy: laboratory tests, modeling, and performance analyses. Applied Thermal Engineering, 205, 118028
[7] Zhao, W, Zhang, Y. Li L, Su, W. Li, B., Fu, Z. Snow melting on the road surface driven by a geothermal system in the severely cold region of china. Sustainable Energy Technologies and Assessments, 2020, 100781.
[8] Xi, J.; Li, Y.; Liu, M.; Wang, R.Z. Study on the thermal effect of the ground heat exchanger of GSHP in the eastern China area, Energy, 2017, 141: 56-65

”

**Comment 3.** *“Jinan (116°E, 37°N) is located in the North region of China, and there are several snowfalls every year” – Please be specific. Provide data on the amount of snowfalls, rather than simply stating that there is 'several.'*

**Reply**:

To take into account the comments and clarify this point, we modified the presentation because the amount of snowfall from year to year in varied and difficult to quantitatively characterize:

“The bridge deck snow melting project relied on in this study is located in Jinan City, Shandong Province of China. Jinan (116°E, 37°N) is located in the North region of China~~, and there are several snowfalls every year~~. The annual average temperature of Jinan is 11°C, and the minimum temperature is approximately −16 °C in winter in past ten years.”

**Comment 4.** *Part “2.1”. The authors constantly talk about GSHPS, but the reader still does not understand what it is. It is recommended to include a technical description.*

**Reply**:

To take into account the comments and clarify this point, we have added the explanation in the section of introduction

“The thermal energy from the ground can be utilized as a heat source in heat pumps (HPs) to reduce the rapid consumption of fossil fuels. the systems of ground source heat pumps (GSHPS), utilizing the geothermal energy are known as geothermal heating pumps that absorb/transfer thermal energy to/from the earth using a series of buried pipes and which can be considered one of the most efficient technologies in the heating/cooling industry”

**Comment 5.** *Part “2.3 The inlet temperature and flow rate” – Why do the authors not use references for the literature? I am certain that the formulas are not the authors' own development.*

**Reply**:

To take into account the comments and clarify this point, we added an important reference for the formula (1)

“The calculation formula [30] is as follows.”

Reference [30]:

[30]	Xi, J.; Li, Y.; Liu, M.; Wang, R.Z. Study on the thermal effect of the ground heat exchanger of GSHP in the eastern China area, Energy, 2017, 141: 56-65

**Comment 6.** *On some figures, the authors use units of temperature in Celsius, while on others, they use Kelvin. Please standardize the units of measurement for consistency.*

To take into account the comments and clarify this point, we modified the relevant figures. The units of temperature in Celsius is adopted.

“

(a) hole spacing 5m

(b) hole spacing 4m

(c) hole spacing 3m”

**Comment 7.** *Figure 12 does not provide any useful information to the engineering and scientific community and is recommended for removal. What information do photos 12(b) and 12(e) convey? The reader only sees that you have some sort of sensor. If the authors believe this information is essential for understanding the paper, a wiring diagram for the sensors is recommended instead of a photo of a twisted wire.*

To take into account the comments, we have delete all the photos.

**Comment 8.** *Figure 15 – To save on printing costs and reduce carbon footprint, many readers print figures in black and white. I cannot analyze this graph effectively because the authors did not use different symbols to distinguish between the various lines.*

To take into account the comments, we have modified the original image that wasn’t friendly. The modified figure is as follow

**Comment 9.** *It is puzzling that the paper completely lacks a Discussion and Results section. This article is missing one-third of the essential information. How do you intend to publish a paper without any discussion of your results?*

To take into account the comments and clarify this point, we have adjusted the structure of the manuscript and added the discussion and results section. The new sections of the manuscript are as follows

“”

Reviewers #2

**Comment 1.** *Revise the abstract carefully. Which finite element software has been employed in the present study? Mention the findings of the study.*

**Reply**:

To take into account the comments, we improved our writing and revised the abstract accordingly. The Finite element software Fluent is also mentioned.

“**Abstract:** Geothermal energy is increasingly employed across diverse applications, with bridge deck snow melting emerging as a notable utilization scenario. In Jinan city, China, a project is underway to utilize ground source heat pumps (GSHPS) for heating bridges. However, essential operational parameters, including fluid medium, temperature, and heat exchange details, are currently lacking. This study addresses the thermal design challenges associated with ground heat exchangers (GHE) for bridge heating through a combination of numerical modeling and field experiments. ~~Unlike traditional utilization of ground source heat pump system (GSHPS) on buildings, bridge heating using geothermal energy in winter have special requirements for fluid media, temperature, and heat extraction.~~ ~~Firstly, based on~~ ~~an engineering case of snow melting bridge deck with GSHPS constructed in Jinan China, A three-dimensional heat transfer model using computational fluid dynamics was built and verified.~~ ~~Then, the controlled variable research method was employed to learn the thermal performance.~~ ~~The influences of the inlet temperature, backfill material, drilling depth, and hole spacing on the heat transfer efficiency of the ground heat exchanger were studied under the alternative various engineering conditions~~. ~~Based on on-site construction testing, the influence of pipeline spacing on bridge deck temperature rise and the temperature variation characteristics of backfill materials were obtained studied~~. Utilizing software Fluent, a refined three-dimensional multi-condition heat transfer numerical analysis was carried out. Field tests based on actual operating conditions were also conducted and the design parameters were verified. The results indicate that an inlet temperature of 5°C and an aqueous solution of ethylene glycol with a mass concentration of 35% as the heat exchange medium are suitable for the GSHPS in Jinan; Moreover, the influence of backfill material and operation time on the heat transfer efficiency was revealed and the suitable material with 10% bentonite and 90% SiO2 was suggested; Finally, based on the influence of the pipe spacing on the heating characteristics of bridge deck, the transition spacing of 0.2m is given for the temperature response of the bridge deck. This comprehensive study contributes valuable insights through simulation and experimental analysis of the thermal environment variation, aiming to advance the development of GSHPS for bridge deck heating in Jinan, China.

”

**Comment 2:** *The abstract mentions “The influences of the inlet temperature, backfill material, drilling depth, and hole spacing on the heat transfer efficiency of the ground heat exchanger were studied under the alternative engineering conditions”. What are the alternative engineering conditions mentioned here? Explain them.*

**Reply**:

To take into account the comments and clarify this point, we have revised the abstract and deleted the description.

**Comment 3:** *Keywords: Arrange the keywords in alphabetical order..*

**Reply**:

To take into account the comments, we have arranged the keywords in alphabetical order.

*“”*

**Comment 4:** *Introduction: Elaborate the introductory part.*

**Reply**:

To take into account the comments, we have elaborated the section of introduction.

“Due to the unfriendly carbon emissions of fossil fuels, the demand for sustainable new energy is gradually increasing. As an effective and green energy source, geothermal energy is gradually used in a variety of building facilities of the world [1-3]. The thermal energy from the ground can be utilized as a heat source in heat pumps (HPs) to reduce the rapid consumption of fossil fuels. the systems of ground source heat pumps GSHPS, utilizing the geothermal energy are known as geothermal heating pumps that absorb/transfer thermal energy to/from the earth using a series of buried pipes and which can be considered one of the most efficient technologies in the heating/cooling industry. Over the last few decades, GSHPS, because of the high-performance factor and more economical operating costs, have acquired significant attention as sustainable alternative energy resources for building cooling/heating applications [4]. ~~To further improve the geothermal utilization in heating system for bridge decks, it is crucial to study the heat transfer capacity of the heat exchangers and obtain the optimal parameters governing its performance.~~

The first interest in the technology of GSHP was known after the Second World War until the early 1950s in North America and Europe. Another significant surge occurred in the 1970s following the global oil crisis, marking a period of increased GSHP activity worldwide.

……

Bridge deicing is critical to ensure roadway safety, mobility, and productivity. Adding salts to melt ice/snow are the most common deicing method but are corrosive to the bridge surfaces and polluting to environment.

……

Previous studies concluded the inlet temperature and flow rate of medium, the thermal conductivity of backfill material, the borehole depth and spacing having different effects on the engineering performance of GSHPS. Although some experimental and simulation analysis on GSHPS had been conducted, ~~due to the different types of pipe, backfill materials and stratum properties,~~ theoretical guidance on the design of GHE with the bridge deck heating conditions has not been unified. To further improve the geothermal utilization in heating system for bridge decks under the specific geological and atmospheric conditions for Jinan of China, it is crucial to study the heat transfer capacity of the heat exchangers and obtain the optimal parameters governing its performance.

”

Some current works:

[9] Hussein M. Maghrabie, Mahrousa M. Abdeltwab, Mohamed Hamam M. Tawfik. Ground-source heat pumps (GSHPs): Materials, models, applications, and sustainability, *Energ. Buildings*, 2023, 299: 113560,
[10] Chen, X.; Kong, G.; Liu, H.; Yang, T.; Zhu, X. Experimental on thermal performance of bridge deck with hydronic heating system. *Cold Reg Sci Technol*, 2020, 178(3): 103130.
[11] Yu, X.; Hurley, M. T.; Li, T.; Lei, G.; Pedarla, A. Puppala, A.J. Experimental feasibility study of a new attached hydronic loop design for geothermal heating of bridge decks. *Appl Therm Eng*, 2020(164):114507
[12] Liu, X.B.; Rees, S.J.; Spitler. J.D. Modeling snow melting on heated pavement surfaces. Part I: Model development. Appl. Therm. Eng., 2007, 27 (5–6): 1115-1124,
[13] Ho, I. H.; Li, S.; Abudureyimu, S. Alternative hydronic pavement heating system using deep direct use of geothermal hot water. *Cold Reg Sci Technol,* 2019. 160(APR.): 194-208.
[14] Lei, G. , Yu, X. , Li, T. , Habibzadeh-Bigdarvish, O. , & Hurley, M. T. . (2022). Insulated pex-pipe loops for deicing on existing bridge deck using geothermal energy: laboratory tests, modeling, and performance analyses. Applied Thermal Engineering, 205, 118028
[15] Zhao, W, Zhang, Y. Li L, Su, W. Li, B., Fu, Z. Snow melting on the road surface driven by a geothermal system in the severely cold region of china. Sustainable Energy Technologies and Assessments, 2020, 100781.
[16] Xi, J.; Li, Y.; Liu, M.; Wang, R.Z. Study on the thermal effect of the ground heat exchanger of GSHP in the eastern China area, Energy, 2017, 141: 56-65

”

**Comment 5:** *It is written that “Many factors affect the thermal performance of ground heat exchanger (GHE), so several studies have been conducted from different perspectives”. What are those factors? Explain them.*

**Reply**:

To take into account the comments, we have added the explanation in section “introduction”:

Many factors affect the thermal performance of ground heat exchanger (GHE), including the type of buried pipes, the backfill materials, the inlet and outlet temperatures, and also the depth of GHE. Thus, several studies have been conducted from different perspectives.

**Comment 6:** *Mention objectives and research significance of the study.*

**Reply**:

To take into account the comments and clarify this point, we have added the content:

“Previous studies concluded the inlet temperature and flow rate of medium, the thermal conductivity of backfill material, the borehole depth and spacing having different effects on the engineering performance of GSHPS. Although some experimental and simulation analysis on GSHPS had been conducted, ~~due to the different types of pipe, backfill materials and stratum properties,~~ theoretical guidance on the design of GHE with the bridge deck heating conditions has not been unified. To further improve the geothermal utilization in heating system for bridge decks under the specific geological and atmospheric conditions for Jinan of China, it is crucial to study the heat transfer capacity of the heat exchangers and obtain the optimal parameters governing its performance.”

**Comment 7:** *What is novelty in the present study?*

**Reply**:

To take into account the comments and clarify this point, we have modified the last paragraph of the section “introduction”

“The study focuses on the heat exchange characteristics of underground and pavement buried pipes with various parameters for an actual bridge deck heating system conducted in Jinan, China. The influence of different inlet temperatures of buried pipes, backfill material ratios and other factors on heat transfer efficiency are investigated through simulation and field test study, which can be used to guide the design and operation of the system. ~~Based on practical engineering, the impact of pipeline spacing on road surface temperature was measured, as well as the temperature evolution of backfill holes during system operation.~~

”

**Comment 8:** *Figure 2 shows Three-dimensional geometric model and the meshing of buried U-type pipe. How the meshing was achieved?*

**Reply**:

To take into account the comments and explain discretization and meshing of pipeline, the following published papers are sited.

[1] “Tahzeeb, R.; Alam, M.; Muddassir, S.M. Blast performance enhancement prediction of circular column with helical mesh reinforcement and strengthened with UHPFRC plaster, and CFRP wrapping under close-in blast. International journal of structural engineering. 2023, 13(2): 221-258. DOI: 10.1504/IJSTRUCTE.2023.130148
[2] Tahzeeb, R.; Alam, M.; Anas, SM; Muddassir, S.M. Dynamic response of CFST column with in-plane cross reinforcement and partial CFRP wrapping upon contact blast. Innovative Infrastructure Solutions. 2023, 8: 241. DOI: 10.1007/s41062-023-01201-x ”

**Comment 9:** *In section 2.2, it is written that “a controlled variable research method was employed,”. How this controlled variable methodology was achieved?*

**Reply**:

To take into account the comments and clarify this point, we changed the description.

“To obtain more accurate engineering design parameters, numerical model analysis under multiple working conditions was carried out ~~a controlled variable research method was employed~~, which involves matching different material and physical parameters and necessary initial boundary constraints to the model.”

**Comment 10:** *Check the citations of the Figures and Tables carefully.*

**Reply**:

We have checked and modified accordingly.

**Comment 11:** *Elaborate the Field test and result.*

**Reply**:

To take into account the comments, we have elaborated the field test and results.

In section 3.1

“The bridge is 28m in length and 25m in width (four car lanes and two sidewalks). From top to bottom, the bridge deck structure consists of a 10cm thick asphalt waterproof layer, a 15cm thick cast-in-place concrete layer, and an 80cm thick precast hollow concrete slab. The construction process of the bridge snow melting project involves several steps, including drilling underground pipes, laying underground and bridge deck pipelines, collecting pipelines, and connecting heat pumps. Holes are drilled into the slope of the bridge head to a depth of 100m, as shown in Figure 3a. ~~The spacing between the pipelines laid on the bridge deck is approximately 30cm, as depicted in Figure 12b.~~ To study the effect of pipe spacing on bridge deck temperature, three different pipe spacing schemes of 0.15, 0.2, and 0.25 were implemented. The heat exchange pipes were tied to the reinforced mesh of the cast-in-place concrete layer and buried 2 cm below the reinforced cement concrete surface. As a result, the pipes were 12 cm beneath the bridge deck surface. The heat exchange pipes beneath the asphalt layer and within the bridge deck were collected and connected to the heat pump system, as illustrated in Figure 3b. All exposed pipelines are insulated using prefabricated polyurethane foam technology, as presented in Figure 3c. Unlike conventional methods for measuring fluid temperature, this test focuses on capturing the temperature variation characteristics of backfill materials during the operational period of the bridge heating system. A temperature measurement line was installed on-site along with the No. 5 buried pipe. The positions of the temperature measurement holes are indicated in Figure 1. ~~The temperature measurement lines consist of series-connected thermocouples, as shown in Figure 12e.~~

In section 4.5

Therefore, it can be determined that the temperature outside the backfill area is uniformly distributed with depth and with time. It also can be seen temperature dropped from 15°C to about 9°C within the 1 day of operation. For comparison, the field test results on temperature distribution of the backfill material over time are also presented. The temperature of soil layers at 2m and 7m from the ground was notably affected by the external atmospheric temperature, resulting in marked differences in the measured temperature of the backfill material compared to the overall curve. The initial temperature of the layers below 10m was approximately 15°C. As the test time for heating the bridge deck increased, the temperature of the backfill material gradually decreased. After 3 hours of testing, the overall backfill material temperature lowered to around 10°C. The measured temperature is slightly higher than the numerical simulation results because the measurement point is located inside the borehole, while the numerical simulation is at the interface between the backfill material and the ground. In addition, the temperature variation of the backfill material at the bottom of the buried pipe was greater than that at the top.”

**Comment 12:** *The result and conclusion parts are very brief. There are only 3 findings and only 1st finding is relevant. 2nd and 3rd findings are part of result, they don’t state any relevant information. The conclusion must include at least 3 findings.*

**Reply**:

To take into account the comments, we have adjusted the structure of the manuscript, and added the part “results and discussion”. The conclusions were modified as below:

“(2) As the heat exchanger spacing expands, the thermal interference effect generated between them gradually diminishes, and the average soil temperature increases, thus enhancing the heat extraction capacity of the heat exchanger. ~~After operating for 8 hours, the relative heat exchange power under the condition of 4m spacing increased by 5.4% compared to that under the condition of 3m spacing, and it increased by 1.9% compared to that under the condition of 5 m spacing.~~ As the operation time progresses, the temperature of the backfill material gradually decreases, and for a 3-hour operation time, the temperature changes around 5°C.

(3) ~~Through on-site installation testing, the influence of different pipeline spacing on bridge deck heating was compared, and~~ The spacing of GHEs plays a pivotal role in determining the temperature gradient between the heat exchangers and the soil, thereby impacting heat exchange efficiency. In the context of the Jinan project in China, a recommended borehole spacing of 4m is advised. Additionally, the density of buried pipes laid on the bridge deck has a notable effect on the evolution of the bridge surface temperature. A critical spacing of 0.2m for laying buried pipes on the bridge deck is identified; beyond this value, the surface temperature shows no significant sensitivity to the spacing. ~~a pipe spacing of 0.2m can meet the heating needs of the bridge deck. As the operation time progresses, the temperature of the backfill material gradually decreases, and within a 3-hour operation time, the temperature changes to around 2°C.~~”
