# Supplementary material for: Heat exchange characteristics of underground and pavement buried pipes for bridge deck heating conditions
Source: PLoS One. 2024 May 14;19(5):e0298077. doi: 10.1371/journal.pone.0298077 (PMC11093364; doi:10.1371/journal.pone.0298077)
Supplement: Attachment — Submitted filename: PONE-D-23-29395 review.docx [file pone.0298077.s004.docx]

The paper requires revision before final publication.

The introductory part is very short. Elaborate it.

Apart from China, most of the parametric studies which has been shown from Fig. 4 to Fig. 8 have been already conducted. What is the advanced novelty in the present study?

It is mentioned that “Although some experimental and simulation analysis on GSHPS had been conducted, theoretical guidance on the design of GHE with the bridge deck heating conditions has not been unified.” But the designing process has not been elaborated here as well.

What are the equations used in the simulation part i.e., heat transfer efficiency at different inlet temperatures? Heat transfer efficiency with different concentrations of media? Heat extraction capacity of different backfill materials? Heat extraction capacity per linear meter at different depths?

It is mentioned that “The heat transfer process between the ground heat exchanger and the surrounding soil is a nonstationary process that involves multi-layer heat transfer media and convection heat transfer. Due to the complex geometry of the 3D buried pipe heat exchanger, certain assumptions and simplifications were made to ensure the accuracy of the established model. These assumptions include: (1) Considering the soil as a consistent and balanced void-free entity. If the soil is assumed be void free, it will act as steel. But the real media is soil, so the assumption fails here. How will you justify this statement?

(2) Assuming that all physical parameters are constant during the heat exchange process; (3) Neglecting the external weather and shallow surface heat exchange, thermal convection, and thermal radiation.” If all physical parameters will be assumed constant, how the study shows real condition?

Fig 6 shows quality concentration. What significance does it have with heat transfer quantity?

At the end of section 4.3, conclusion has been written. Then what is the meaning of repeating same statement in the conclusion part?
