# Supplementary material for: Heat exchange characteristics of underground and pavement buried pipes for bridge deck heating conditions
Source: PLoS One. 2024 May 14;19(5):e0298077. doi: 10.1371/journal.pone.0298077 (PMC11093364; doi:10.1371/journal.pone.0298077)
Supplement: Attachment — Submitted filename: Response to Reviewers.docx [file pone.0298077.s005.docx]

Reply to reviewers’ comments

The authors gratefully acknowledge the constructive comments from the Reviewers. The revised manuscript was prepared in order to address all the comments, as described in details below. Major modifications are highlighted in the revised manuscript using yellow color for easy reference.

Reviewers #2

**Comment 1.** *The introductory part is very short. Elaborate it..*

**Reply**:

Thank you very much for your constructive comments. We have elaborated on snow/ice melting and deicing technology from the hazards of snow/ice on transportation to maintenance methods. Some revision are as follows:

“During winter, over 70% of roads in China experience ice and snow conditions, leading to significant challenges that impact the safety, mobility, and productivity of the transportation system [1]. Snow accumulation can pose serious threats, causing reduction of the adhesion coefficient between vehicle tires and the road surface. This reduction contributes to issues such as vehicle slippage, deviation, and extended braking distances, posing risks to the personal safety of drivers and passengers. In the United States alone, each year witnesses over 1300 fatalities and more than 116,800 injuries resulting from vehicle crashes on snowy, slushy, or icy pavement [2]. Over $6 and $10 billion are spent on the snow and ice removal annually in the USA and China, respectively. This cost has continued to increase in recent years [3].

Currently, passive methods, including mechanical and chemical snow melting approaches, are primarily employed for ice and snow removal [4][5]. Mechanical methods often involve lane closures or traffic disruption, leading to inefficient snow removal and incomplete clearance. Additionally, snow removal agents have the potential to corrode steel bars in cement concrete pavement or bridge structures, impacting road and bridge durability. Moreover, these agents contribute to environmental pollution when discharged into water and soil. The reliance on passive measures falls short of meeting the demands for green, energy-saving, environmentally friendly, and highly efficient solutions [4]. The thermal snow melting method is recognized as an active, environmentally friendly technology that harnesses heat from various sources[3], including hot water, solar energy, geothermal energy, and electricity, to facilitate snow melting. This method encompasses cable heating, conductive concrete heating, and circulating thermal fluid heating technologies. Cable heating and conductive concrete heating directly rely on electrical energy, offering stability but exhibiting limitations in energy utilization efficiency.

And the added references:

[1] “Xi, J.; Li, Y.; Liu, M.; Wang, R.Z. Study on the thermal effect of the ground heat exchanger of GSHP in the eastern China area, *Energy*, 2017, 141: 56-65
[2] Han, C.; Yu, X.; Feasibility of geothermal heat exchanger pile-based bridge deck snow melting system: A simulation based analysis. *Renew. Energ.* 101, 2017, 214-224.
[3] Zou, J.L.; Gao, Y.S., Liu, Q.; Sun, X.Y. Feasibility of a snow-melting membrane roof with an electrical–thermal system and prediction of its performance. *Appl Therm Eng*. 2023, 222: 119869
[4] Matejicka, L., Georgakis, C.T. A review of ice and snow risk mitigation and control measures for bridge cables. *Cold Reg Sci Technol*, 2022, 193: 103429
[5] Trenouth, W.R., Gharabaghi, B., Perera, N. Road salt application planning tool for winter de-icing operations. *J Hydrol*, 2015, 524:401-410.

”

**Comment 2.** *Apart from China, most of the parametric studies which has been shown from Fig. 4 to Fig. 8 have been already conducted. What is the advanced novelty in the present study? It is mentioned that “Although some experimental and simulation analysis on GSHPS had been conducted, theoretical guidance on the design of GHE with the bridge deck heating conditions has not been unified.” But the designing process has not been elaborated here as well.*

**Reply**:

Thanks to your comments. Yes, there have been reports on bridge snow melting and deicing technology based on GSHPS. The innovation of this article lies in the development and verification of bridge snow melting technology based on the environmental and geological conditions of Jinan. For the designing process, we added the calculation process of snowmelt load.

“The determination of heating requirement for snow/ice removal, which is normally described by surface heat flux, provides important design capacity of the whole system. The required heat flux for snow melting on the basis of steady-state energy balance is given by [31][37]:

$q_{0}=q_{1}+q_{2}+q_{3}+q_{4}-q_{5}$ (A1)

where, *q*_0_ denotes to heat flux required at snow melting surface, W/m^2^; *q*_1_ is the heat flux required to raise the temperature of snow/ice, W/m^2^:

$q_{1}=\rho_{w}S\left[C_{w}(t_{0}-0)+C_{snow}\left(0-t_{a}\right)\right]$ (A2)

*q*_2_ is the latent heat of snowmelt W/m^2^:

$q_{2}=\rho_{w}\times S\times H_{if}$ (A3)

*q*_3_ is the long-wave radiant heat, W/m^2^:

$q_{3}=h_{c}(t_{0}-t_{a})+\varepsilon C_{b}\left[\left(\frac{t_{\text{a}}+273}{100}\right)^{4}-\left(\frac{t_{a}+273}{100}\right)^{4}\right]$ (A4)

*q*_4_ is the heat flux required for convection heat transfer, W/m^2^:

 (A5)

*q*_5_ is the solar radiant heat, W/m^2^:

$q_{5}=\alpha I$ (A6)

The concepts and the values of the above symbols are listed in Table 4.

| Symbols | Concepts | Values | Unit |
| --- | --- | --- | --- |
| *ρ*_w_ | Water density | 1000 | kg/m^3^ |
| *S* | Snowfall speed | 2.5 | mm/h |
| *C*_w_ | Specific heat of water | 4.2 | KJ/(kg·℃) |
| *C*_snow_ | Specific heat of snow | 2.1 | KJ/(kg·℃) |
| *t*_0_ | The temperature of the snow water | 0 | ℃ |
| *t*_a_ | Air temperature | -3.2 | ℃ |
| *H*_if_ | Latent heat of snowmelt | 334 | KJ/kg |
| *h*_c_ | Convective heat transfer coefficient | 17.79 | W/(m^2^⋅K) |
| *C*_b_ | Stefan-Boltzmann | 5.67 | W/(m^2^⋅K^4^) |
| *ε* | Pavement emissivity for asphalt pavement | 0.96 | - |
| *H*_f_ | Latent heat of vaporization of water | 2502c | KJ/kg |
| *ϕ* | Relative humidity of the air | 45 | % |
| *ρ*_wa_ | Saturated water vapor density at 0 ℃ | 0.00485 | kg/m^3^ |
| *h*_m_ | The rate of mass transfer when water evaporates | 0.68 | m/s |
| *α* | Surface solar radiation absorption | 0.6 | - |
| *I* | The amount of solar radiation on the Earth's surface | 182.52 | W/m^2^ |

”

**Comment 3.** *What are the equations used in the simulation part i.e., heat transfer efficiency at different inlet temperatures? Heat transfer efficiency with different concentrations of media? Heat extraction capacity of different backfill materials? Heat extraction capacity per linear meter at different depths?*

**Reply**:

Thanks to your comments. The description of heat exchange efficiency in the previous manuscript was somewhat confusing. To take into account the comments and clarify this point, we have modified the relevant contents as

“it is used here as the evaluation index of heat exchange efficiency. The calculation formula [1] is as follows.

$$Q=\rho c_{p}Au\left|t_{in}-t_{out}\right|$$

$$q=\frac{Q}{L}$$

where *Q* is the total heat exchange, kW, *q* is the heat exchange rate per unit length, W/m; *ρ* is the fluid density, taking the density of ethylene glycol aqueous solution as 1050 kg/m^3^; *c_p_* is the mass-specific capacity of the fluid, 1550 J/(kg·K); *A* is the cross-sectional area within the buried pipe, m^2^, Take the cross-sectional area inside the DN40 pipe as 0.0008m^2^; *u* is the fluid flow rate, m/ s; *t_in_* is the fluid medium inlet temperature, °C; *t_out_* is the outlet temperature, °C. The coefficient of performance of the GSHPS is typically denoted as

$$COP=\frac{Q}{W}$$

where, *W* is the power consumption of the system, kW. Given the fixed power of the heat pump system, the total heat exchange serves as an indicator of the overall system's heat exchange efficiency

Furthermore, we modified the expressions of heat exchange efficiency, especially for the Figures.

**Comment 4.** *Considering the soil as a consistent and balanced void-free entity. If the soil is assumed be void free, it will act as steel. But the real media is soil, so the assumption fails here. How will you justify this statement? If all physical parameters will be assumed constant, how the study shows real condition?*

**Reply**:

Thanks to your comments. Model assumptions are not expressed rigorously. To take into account the comments and clarify this point, we modified the assumption to make the expression more rigorous.

“The heat transfer process between the ground heat exchanger and the surrounding soil is a non-stationary process that involves multi-layer heat transfer media and convection heat transfer. ~~Due to the complex geometry of the 3D buried pipe heat exchanger, certain assumptions and simplifications were made to ensure the accuracy of the established model.~~ To accommodate the intricate geometry of the 3D buried pipe heat exchanger and the limited operating time of heating systems, specific assumptions and simplifications are introduced to uphold the accuracy of the established model. These assumptions include: (1) Considering the soil as a solid material;~~consistent and balanced void-free entity; (2) Assuming that all physical parameters are constant during the heat exchange process;~~ (2) Neglecting the external weather and shallow surface heat exchange, thermal convection, and thermal radiation; (3) Not considering heat exchange related to underground water flow.”

**Comment 5.** *Fig 6 shows quality concentration. What significance does it have with heat transfer quantity?*

**Reply**:

Thanks to your comments. From original Fig 6, the selection of different concentrations does not affect the overall heat transfer exchange efficiency of the system. However, this result makes it reasonable to select the concentration of the medium from the perspective of antifreeze. To clarify this point, we delete the section 4.2 including the Figure 6. In the section of “field test procedure”, we discussed the chosen of the material.

“Research has shown that the medium concentration of heat pump circulating fluid has little effect on the heat exchange rate. Table 2 shows the physical parameters of ethylene glycol aqueous solutions with different mass concentrations. For every 5% difference in mass concentration, the solution has a significant difference in freezing point and viscosity, while the difference in thermal physical parameters is small. In the field test, an aqueous solution of ethylene glycol with a mass concentration of 35% as the heat exchange liquid medium.”

**Comment 6.** *At the end of section 4.3, conclusion has been written. Then what is the meaning of repeating same statement in the conclusion part?*

**Reply**:

To take into account the comments, we have modified the last conclusion at the end of section 4.3

Therefore, ~~it can be concluded that~~ it seems that thermal conductivity is a more important constraint on the heat ~~transfer~~ exchange efficiency than specific heat capacity. ~~Thus, sand containing 10% bentonite and 90% SiO~~_~~2~~_ ~~is the most suitable backfill material for the project.~~

Reviewers #3

**Comment 1.** *It appears that the heat exchangers are coupled with a heat pump, I suggest modifying the title to account for this. Just ‘heat exchanger’ can be confusing.. In the introduction, add ‘ …heat source and heat sink in heat pumps….’*

**Reply**:

Thanks to your comments. For technologies that involve heat flow cycles, heat pumps are generally included. Considering the research content of this manuscript, we adhere to the original title. The relevant expression in the introduction is modified.

“The thermal energy from the ground can be utilized as a heat source and heat sink in heat pumps (HPs) to reduce the rapid consumption of non-clean energy ~~fossil fuels~~ and increase the electricity energy utilization efficiency.”

**Comment 2:** *You still have typos, see line 4 on page 3, the sentence should start with an uppercase letter.*

**Reply**:

Thanks to your comments. We have modified the typo and checked throughout the manuscript.

**Comment 3:** *The first paragraph addresses performance and operation costs, it does not mention anything on the high capital costs of heat pumps?.*

**Reply**:

Thanks to your comments. In the introduction, we have added the part of the hazards of snow/ice roads as well as de-icing technology. From construction to long-term operation for the bridges and highways in the suburbs of the city, the comprehensive cost of heat pumps seems the most cost-effective. The added introduction as

“During winter, over 70% of roads in China experience ice and snow conditions, leading to significant challenges that impact the safety, mobility, and productivity of the transportation system [1]. Snow accumulation can pose serious threats, causing reduction of the adhesion coefficient between vehicle tires and the road surface. This reduction contributes to issues such as vehicle slippage, deviation, and extended braking distances, posing risks to the personal safety of drivers and passengers. In the United States alone, each year witnesses over 1300 fatalities and more than 116,800 injuries resulting from vehicle crashes on snowy, slushy, or icy pavement [2]. Over $6 and $10 billion are spent on the snow and ice removal annually in the USA and China, respectively. This cost has continued to increase in recent years [3].

Currently, passive methods, including mechanical and chemical snow melting approaches, are primarily employed for ice and snow removal [4][5]. Mechanical methods often involve lane closures or traffic disruption, leading to inefficient snow removal and incomplete clearance. Additionally, snow removal agents have the potential to corrode steel bars in cement concrete pavement or bridge structures, impacting road and bridge durability. Moreover, these agents contribute to environmental pollution when discharged into water and soil. The reliance on passive measures falls short of meeting the demands for green, energy-saving, environmentally friendly, and highly efficient solutions [4]. The thermal snow melting method is recognized as an active, environmentally friendly technology that harnesses heat from various sources[3], including hot water, solar energy, geothermal energy, and electricity, to facilitate snow melting. This method encompasses cable heating, conductive concrete heating, and circulating thermal fluid heating technologies. Cable heating and conductive concrete heating directly rely on electrical energy, offering stability but exhibiting limitations in energy utilization efficiency.”

**Comment 4:** *Reading the introduction, I can not find the research gaps that this study intends to fill. I believe the heat transfer capacity of heat exchangers have been studied before*

**Reply**:

Thanks to your comments. The innovation of this manuscript lies in the development and verification of bridge snow melting technology based on the environmental and geological conditions of Jinan. For the designing process, we added the calculation process of snowmelt load.

“The determination of heating requirement for snow/ice removal, which is normally described by surface heat flux, provides important design capacity of the whole system. The required heat flux for snow melting on the basis of steady-state energy balance is given by [31][37]:

$q_{0}=q_{1}+q_{2}+q_{3}+q_{4}-q_{5}$ (A1)

where, *q*_0_ denotes to heat flux required at snow melting surface, W/m^2^; *q*_1_ is the heat flux required to raise the temperature of snow/ice, W/m^2^:

$q_{1}=\rho_{w}S\left[C_{w}(t_{0}-0)+C_{snow}\left(0-t_{a}\right)\right]$ (A2)

*q*_2_ is the latent heat of snowmelt W/m^2^:

$q_{2}=\rho_{w}\times S\times H_{if}$ (A3)

*q*_3_ is the long-wave radiant heat, W/m^2^:

$q_{3}=h_{c}(t_{0}-t_{a})+\varepsilon C_{b}\left[\left(\frac{t_{\text{a}}+273}{100}\right)^{4}-\left(\frac{t_{a}+273}{100}\right)^{4}\right]$ (A4)

*q*_4_ is the heat flux required for convection heat transfer, W/m^2^:

 (A5)

*q*_5_ is the solar radiant heat, W/m^2^:

$q_{5}=\alpha I$ (A6)

The concepts and the values of the above symbols are listed in Table 4.

| Symbols | Concepts | Values | Unit |
| --- | --- | --- | --- |
| *ρ*_w_ | Water density | 1000 | kg/m^3^ |
| *S* | Snowfall speed | 2.5 | mm/h |
| *C*_w_ | Specific heat of water | 4.2 | KJ/(kg·℃) |
| *C*_snow_ | Specific heat of snow | 2.1 | KJ/(kg·℃) |
| *t*_0_ | The temperature of the snow water | 0 | ℃ |
| *t*_a_ | Air temperature | -3.2 | ℃ |
| *H*_if_ | Latent heat of snowmelt | 334 | KJ/kg |
| *h*_c_ | Convective heat transfer coefficient | 17.79 | W/(m^2^⋅K) |
| *C*_b_ | Stefan-Boltzmann | 5.67 | W/(m^2^⋅K^4^) |
| *ε* | Pavement emissivity for asphalt pavement | 0.96 | - |
| *H*_f_ | Latent heat of vaporization of water | 2502c | KJ/kg |
| *ϕ* | Relative humidity of the air | 45 | % |
| *ρ*_wa_ | Saturated water vapor density at 0 ℃ | 0.00485 | kg/m^3^ |
| *h*_m_ | The rate of mass transfer when water evaporates | 0.68 | m/s |
| *α* | Surface solar radiation absorption | 0.6 | - |
| *I* | The amount of solar radiation on the Earth's surface | 182.52 | W/m^2^ |

”

**Comment 5:** *Given the location of the project, the annual average temperature and the ground temperature of 15℃, I am not sure how severe the winter is to require such a complex system that would work a fraction of the time in a year. An economic analysis of this and alternatives should be presented.*

**Reply**:

Thanks to your comments. We added some information of the project in the end paragraph of introduction as

“Jinan (116°E, 37°N), capital of Shandong Provence of China, is located in a hilly area, with several highways passing through the area and multiple bridges constructed. To reduce the impact of snowfall on transportation in winter, it is planned to eliminate snow accumulation through GSHPS.”

And in the part of section 2.1, we deleted the confusing 11°C.

~~The annual average temperature of Jinan is 11 °C~~

In addition, as introduced in the first paragraph, this work is very necessary. However, its economic analysis is not within the scope of this study.

**Comment 6:** *At what moisture content were the properties taken, weighted average values will be influenced by the moisture. Also, are these average values over different seasons?*

**Reply**:

To take into account the comments and clarify this point, we have added the explanations:

“…physical parameters around the heat exchanger were obtained from borehole interpretation data for winter surveys. On average,…”

**Comment 7:** *The load is presented in a simplistic way, the equations used to determine this can either be in the appendix or in the paper.*

**Reply**:

Thanks to your comments. To take into account the comments, the calculation process is presented in the appendix, which can be seen in the Comment 4.

**Comment 8:** *The extraction rate depends on several factors and can change with season and over time. Also, when you write units, leave a space between the number and the unit. i.e. 80 m and not 80m, except for percent and degree C.*

**Reply**:

To take into account the comments, we have modified all the writing problem.

**Comment 9:** *Page 7, ‘….the minor factors that considered….’ is a type*

**Reply**:

To take into account the comments and clarify this point, we changed deleted the ambiguous word

“The ~~main~~ factors that considered include the inlet medium temperature and flow rate of the heat exchanger…”

**Comment 10:** *The CFD methodology is lacking in may respects, it is not clear what the flow regime is, what governing equations are solved and how. The validation of the model and the mesh dependency tests.*

**Reply**:

To take into account the comments, we have added the governing equations.

The governing equations for the non-isothermal pipe flow numerical model are described as follows:

$$\frac{\partial\rho_{\text{w}}}{\partial t}+\text{div}\left(\rho_{\text{w}}u\right)=0$$

$$\frac{\partial\left(\rho_{\text{w}}u\right)}{\partial t}+\text{div}\left(\rho_{\text{w}}u\right)=\text{div}\left(\mu\text{grad}u\right)-\text{div}\left(P\right)+F$$

$$\frac{\partial\left(A\rho_{\text{w}}T\right)}{\partial t}+\text{div}\left(A\rho_{\text{w}}uT\right)=\text{div}\left(A\frac{k}{C_{p}}\text{grad}T\right)+S_{T}$$

where, *ρ*_w_ is the density of the warer, kg/m^3^. *u* is the fluid velocity, m/s. P is the pressure in N/m^2^. F represents the volume force in N/m^3^. *C*_p_ is the heat capacity at constant pressure in J/(kg·K). *T* is the temperature in Kelvin. k is the thermal conductivity in W/(m·K), *S*_T_ is a source/sink term accounting for heat exchange with the surroundings through the pipe wall in W/m. A is the cross section area of the pipe in m^2^. Equations (1)-(3) describes the heat transfer in the circulating ﬂuid, encompassing conduction, convection, and dissipation, as well as the heat transfer between circulating ﬂuid and the pipe wall (via the source/sink term *S*_T_).

For the validation of the model, we initially validated an experiment based on Chen et al. (2021)'s experimental results, as shown in the Figure R1. The comparison indicates the reliability of the model. Considering the focus of the manuscript, this comparative process was not expressed and only the following modifications have been made in the text.

Fig. R1 Validation of the model

Refereence

Chen, Z.; Yao, J W.; Pan, P.; Xiao, H.; Ma, Q. Research on the heat exchange characteristics of the deeply buried pipe type of energy pile. Case. Stud. Therm. Eng. 2021. 101268.

The modifications are “Through reliability verification, the model can be well used to simulate the temperature field analysis of GSHPS. The backfill area and fluid domains are meshed with sufficient density to meet the calculation accuracy requirements with the aid of ICEM software”

**Comment 11:** *Page 10, 40 KW, should be 40 kW, unit for power! Change this even in the results section. How was the flow rate for the fluid selected?*

**Reply**:

To take into account the comments, we have modified the typos in the texts and figures. For the flow rate of the fluid, it is calculated by total flow rate and cross-sectional area of pipes, rather than selected. To clarify this point, we have changed the relevant wording:

“Since each host heat source will be connected to a 5-way branch port size DN40 manifold. It is easy to ~~get~~ calculate the buried pipe inlet flow rate of 0.7 m/s.”

**Comment 12:** *In addition to the numerical modelling procedure, the testing itself needs further explanation. What are the uncertainties in the individual sensors? Then present a detailed and expanded uncertainty analysis.*

**Reply**:

To take into account the comments, we have added the explanation of the test monitor system as

“The essential temperature measurement equipment comprises an AC voltage regulator, a resistance thermometer sensor, field data acquisition, and a data adapter. The resistance thermometer sensor has a measurement range of -50 to 150°C with an accuracy of 0.05°C. The field data acquisition is employed to gather temperature signals, which are then connected to the data adapter. The monitoring system automatically stores the temperature data obtained in the computer system.”

**Comment 13:** *Look at the recommendations for spacing of borehole heat exchangers, I do not think the comparison is reasonable when the literature suggests a larger separation to avoid thermal interference.*

**Reply**:

To take into account the comments and clarify this point, we have modified the explanation as

“Generally speaking, the operation of GSHPS for the bridge snow melting condition is only a few days. To highlight the effects of different borehole spacing”

And

“At 8 h, heat exchange increases by 5.4% under 4 m spacing compared to 3 m spacing, while it increases by 1.9% under 5 m spacing compared to 4 m spacing. Therefore, from the perspective of effective utilization of the ground, the optimal borehole spacing of 4 m will be better in the project.”

**Comment 14:** *I do not think section 4.2 should be in the results section, since this is your materials. You can discuss this in the section of your field tests. If you data for this, you present the increase or decrease in performance.*

**Reply**:

To take into account the comments, we have deleted the 4.2 section and added some discussion in the section of field test.

“Research has shown that the medium concentration of heat pump circulating fluid has little effect on the heat exchange rate. Table 2 shows the physical parameters of ethylene glycol aqueous solutions with different mass concentrations. For every 5% difference in mass concentration, the solution has a significant difference in freezing point and viscosity, while the difference in thermal physical parameters is small. In the field test, an aqueous solution of ethylene glycol with a mass concentration of 35% as the heat exchange liquid medium.”
